# Torques in the human upper ankle joint level and their importance in conservative and surgical treatment

**DOI:** 10.1038/s41598-024-57698-4

**Published:** 2024-03-29

**Authors:** Jacek Dygut, Monika Piwowar

**Affiliations:** 1 KAL-Med Consulting, Broom House Quarrywood Court, Livingston, EH54 6AX Scotland; 2 Department of Bioinformatics and Telemedicine, Faculty of Medicine, Jagiellonian University Medical College, Kopernika 7e St., 31-034 Kraków, Poland

**Keywords:** Muscle torque, Upper ankle joint, Biomechanics of the foot, Moment of the force, Force of the extrinsic muscles, Arm of the force, Skeletal muscle, Tendons, Mechanical engineering, Biomedical engineering

## Abstract

The paper deals with the torques of external muscles acting on the upper ankle joint under weight-bearing conditions and their importance in diagnosing and treating the human foot. Experimental data were collected and calculations were performed. Based on the experiments with the biomechanical model of the foot and upper ankle joint, it was shown how the changes in the force arms of the external muscles of the foot under weight-bearing conditions, change the torque. The real values of muscle forces and torques of the external muscles of the foot were calculated. Taking into account the distance of the lines of muscle action from the axis of rotation of the upper ankle joint the rotational force of the muscles was calculated. The influence of changing the force arm on the rotational efficiency of the muscle balancing the moment of gravity was shown. Knowledge of muscle torque under weight-bearing conditions is crucial for correctly assessing foot biomechanics. It has been shown that torque (gravitational and muscular), not pure force, is crucial when assessing the rotational capacity of the analyzed joint. A change in the approach to diagnostics and treating paresis or weakness of extrinsic foot muscles was proposed through the manipulation of the distance of their action line from the axis of joint rotation.

## Introduction

In the professional literature, the function of the muscles acting on the foot in non-weight-bearing conditions is usually described. Then these characteristics are transferred to the conditions of the weight-bearing foot. This approach often leads to erroneous conclusions, which influences the developed therapies and treatment effects^1,2^.

When muscles are characterized, the muscle mass is most often treated as an exponent of muscle strength. It is not taken into account that the strength of a muscle is determined by its physiological cross-section (PCSAand not its muscle mass alone.

Another common error in the description of the action of foot muscles on the foot joints is taking into account the force of muscle contraction as the only factor resulting in the rotational movement of the foot joints^1,2^. Most surgical procedures correcting incorrect positioning of the foot are based only on the assessment of the strength of muscle contraction, without taking into account the distance of the action line of the muscle from the joint rotation axis (arm of the force)^1–4^.

A muscle without an arm doesnot create rotational motion on the joint^16^. The rotational movement of the foot joints is generated by muscle force acting with a distance from the joint rotation axis (arm of the force), thus generating moments of muscle forces, i.e. torques^5–9^. The importance of the action of muscles through its force arm is demonstrated by the presence of numerous anatomical structures in the foot structure, i.e. sesamoids (movable lever support points) or immovable muscle trochlea^9–11^ and the medial and lateral ankle, whose task is to move the line of action of the muscles away from the axis of joint rotation, causing the creation or increase of torques of these muscles. The purpose of such structures is to minimize the energy expenditure of muscles permanently overcoming the force of gravity both while standing and walking. Therefore, in muscle analysis, in addition to the force of muscle contraction, the moments of muscle forces are important. If the muscle rotational capacity deviates from the norm, it indicates two possibilities. The first is a reduction in muscle strength of neurogenic origin (e.g. paresis. paralysis) or myogenic origin (e.g. dystrophy, atrophy, inflammation, and muscle injuries). The second is a change in the length of the muscle force arm (e.g. post-traumatic and post-operative scar changes, disruption or cutting of retinaculum, improperly healed avulsion fractures, removal of trochlea and sesamoids).

Since the human body is subject to the Earth's gravitational field when analysing the action of the foot muscles under weight-bearing, it is important to take into account, in addition to the moments of muscle forces, and the moment of gravity. The state of balance at the upper ankle joint level is achieved by constantly balancing the moments of muscle forces on both sides of the joint rotation axis (action of antagonists and agonists) and the moment of gravity^12^. Too much load on the foot, e.g. due to obesity (increases the force of gravity) or posture deformities (increases arm the force of gravity) increases the gravitational torque that deforms the foot.

In the professional literature concerning the human body, there are only a few publications in which, in addition to muscle strength, the importance of the arm of the force is described^13–15^. In the foot biomechanics description, there are also few of them^3,6,7^. In only a few publications the balance between the moment of the muscle force and the moment of gravity in the weight-bearing foot is considered^5,9,16–18^. Probably, that knowledge was not translated into common medical practice, due to a lack of data concerning the real force values of the extrinsic muscles and torques for joints of the foot. It is crucial for understanding muscle functions and designing effective therapeutic methods, both conservative and surgical. Examples of surgical procedures in which knowledge about forces and moments of force are necessary for proper operation aree.g. hallux valgus, transversely flat foot, and plano-valgus foot. Knowledge about the rotational abilities of individual joints, as well as knowledge about muscle forces and force arms will allow specialists to precisely develop the course of the procedure, thus adapting it to a specific patient. It will also be useful in planning rehabilitation and conservative treatment.

The paper is an attempt to fill the gap in knowledge regarding muscle forces and moments of force generated by the foot muscles as well as the influence of the moment of gravity on the muscular system of the human foot. Research on the model of the foot and upper ankle joint provided answers to the following questions:

- Where are the arms of muscular forces located and what significance do they have for the real rotational force of the muscle?
- What is the mechanism for balancing the body leaning forward and backward about the upper ankle joint?
- How by increasing the arm of the forces, the paresis or weakness of the main flexor as the triceps surae muscle or the main extensoras the tibial anterior muscle be compensated?

## Results

### Forces and torques produced by the extrinsic muscles of the foot

Physiological cross-sectional area (PCSA), maximum force (F_m_), arm of the force was calculated. Having data on the force of the muscle and the distance of its line action (arm of force) from the axis rotation of the upper ankle joint, the torques developed by the foot muscles were calculated (Table 1).

**Table 1:** The biomechanical properties of extrinsic muscles of the upper ankle joint in human.

Muscle	Muscle Mass [g]	Fiber length [cm]	Pinnation Angle [Θ]	Cos(Θ)	PCSA [cm2]	F_m_ [kG]	F_m_ [N]	r_m_ [m]	M_m_ [kGm]	M_m_ [Nm]
Tibial anterior (TA)	65.7	7.73	5°	0.996	8.01	80.16	786.41	0.03	2.4	23.59
Fibularis longus (PL)	41.5	3.87	10°	0.984	9.99	99.92	980.25	0.015	1.5	14.7
Tibial posterior (TP)	53.5	2.4	11.7°	0.979	20.66	206.66	2027.36	0.01	2.07	20.27
Fibularis brevis (PB)	17.3	3.93	5°	0.996	4.15	41.52	407.3	0.015	0.62	6.11
Fibularis tertius (FT) *	–	–	–	–	2.85	28.47	279.32	0.025	0.71	6.97
Flexor digitorum longus (FDL)	16.3	2.7	6.7°	0.993	5.67	56.77	556.9	0.015	0.85	8.35
Flexor hallucis longus (FHL)	21.5	3	10°	0.984	5.89	58.92	578.04	0.025	1.47	14.45
Extensor digitorum longus (EDL)	35.2	8.03	8.3°	0.989	4.1	41.05	402.74	0.025	1.03	10.07
Extensor hallucis longus (EHL)	12.9	8.7	6°	0.994	1.39	13.96	136.92	0.03	0.42	4.11
soleus (SO)	215	1.95	25	0.906	94.59	945.95	9279.77	0.04	37.84	371.19
gastrocnemius medialis (GM)	150	3.53	16.7	0.958	38.55	385.49	3781.7	0.04	15.42	151.27
gastrocnemius lateralis (GL)	96	5.07	8.3	0.989	17.73	177.33	1739.66	0.04	7.09	69.59

PCSA, physiological cross-section; F_m_, maximum force of the muscle; r_m_, the arm of the force; M_m_, maximum torque of the muscle (moment of the force). The force is in two units, i.e. kilogram-force [kG] and newton [N]. The torque is in kilogram-meter [kGm] and newton-meter [Nm], arm of the force is in meter [m].

*The PCSA and force of the m. fibularis tertius was calculated based on the value of the relative strength (Silver et al.^1^) by proportionality to the forces of other muscles due to the lack of mass, length, and pinnation angle data of the m. fibularis tertius.

In the human foot, there is a 14.6-fold advantage in the moments of force of the flexors (SO, GM, GL, TP, FDL, FHL, FL, PB) over the extensors (TA, EHL, EDL, FT) of the upper ankle joint (Table 1). The ratio of the absolute sum of flexor forces over extensors is 12.

### Balancing the body weight leaning forward by the torques of the flexor muscles

Examples of balancing the moment of gravity by external muscles of the foot in a situation similar to physiological conditions and when their lines of action are distancing from the axis of rotation of the upper ankle joint are presented below.

#### Relationship between the triceps surae strength and its arm of the force on the upper ankle joint level

When the center of gravity is leaned forward from the axis of the upper ankle joint, the inserts of the triceps surae (TRI) move away, the lamina pedis approaches the lower leg (extended position) and TRI is in eccentric contraction. The extensor group muscles are not involved in balancing such a system. They are only in compensatory concentric contraction to ensure smooth movement.

To achieve balance in the model system when the lower leg is leaned forward, the gravitational moment (M_G_ = F_G_*r_G_) must be balanced with the muscular moment (M_TRI_ = F_TRI_*r_TRI_).

**Figure 1:**
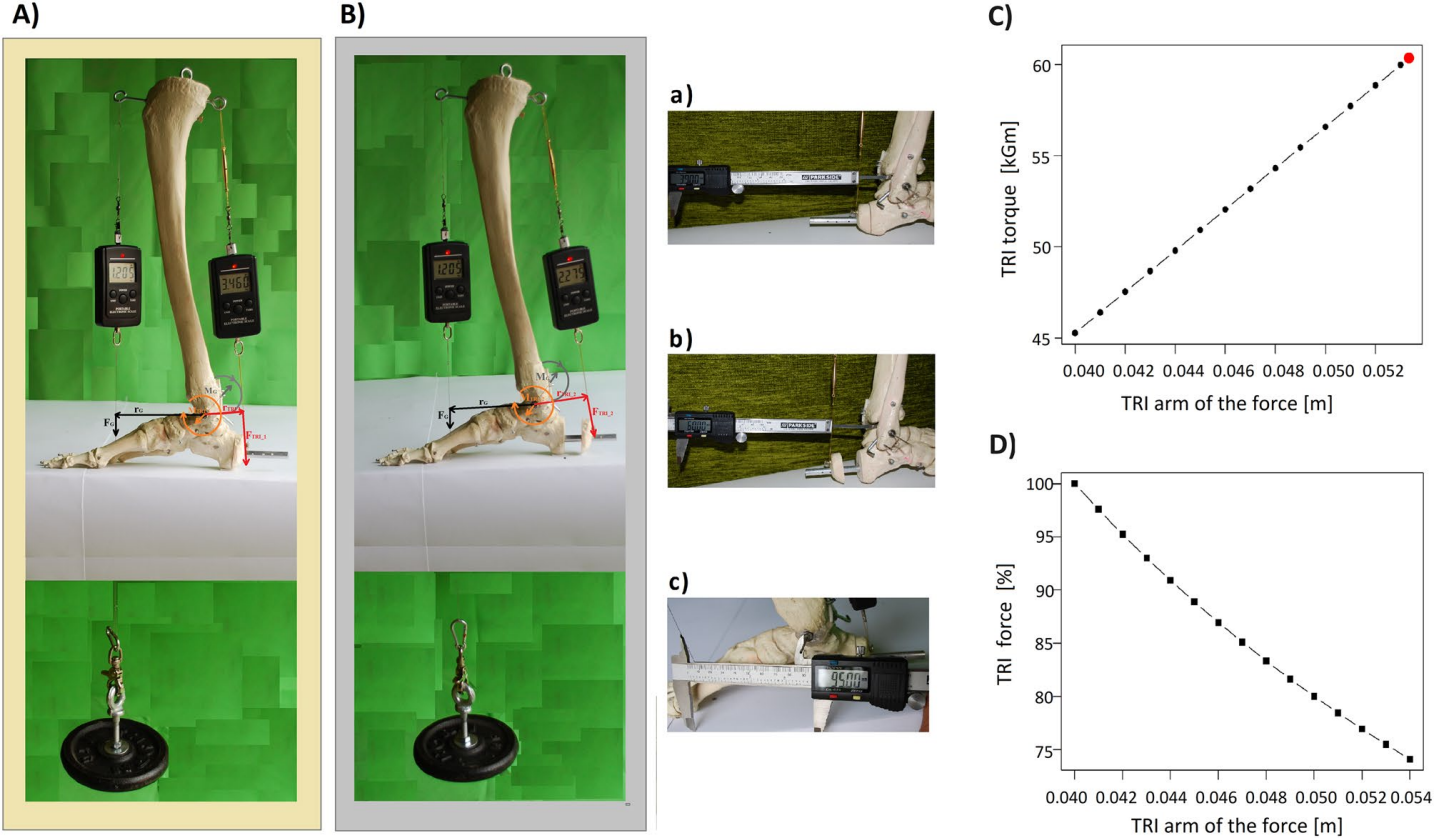
Balancing the moment of gravity by the triceps surae (TRI) torque. The strength (force) of TRI at a constant value of the force of gravity (F_G_ = 1.205 kG + 0.225 kG) and moment of gravity (M_G_ = F_G_*r_G_ = 0.1358 kGm) on the upper ankle joint level. (**A**) in conditions similar to physiological ones, in which the arm of force for the TRI is 0.039 m (r_TRI_1_), (F_TRI_1_ = 3.46 kG). (**B**) in the case of increasing the arm of the force of the TRI by moving the tuber of the calcaneal bone (and its insert) away on the guide to 0.06 m (r_TRI_2_), (F_TRI___2_ = 2.275 kG). (**C**) The relationship between the torque and the length of the arm of the force at a constant value of the force of the TRI muscle weakened by 25%. As the arm grows, the torque increases to a value close to the physiological value (red dot). (**D**) The relationship between the force of the muscle and the length of the arm of force at a constant torque of a TRI muscle weakened by 25%. The more weakened muscle the length of the arm of the force proportionally longer to maintain the physiological torque. (**a**) Measurement of the arm of the force of TRI without the distance (r_TRI_1_ = 0.039 m) and (**b**) with the distance (r_TRI_2_ = 0.06 m). c) Measurement of the arm of gravity (r_G_ = 0.095 m).

In conditions similar to physiological ones, when the arm of the force of TRI is 0.039 m (Fig. 1a) (instead of 0.04 because the model has a millimeter defect in the calcaneus after cutting—a procedure necessary to move the calcaneal bone on the guide), the arm of the force of gravity is 0.095 m (Fig. 1c), and the force of gravity of the weight-bearing the leaning lower leg with a dynamometer is 1.43 kG (F_G_ = 1.205 kG + 0.225 kG), then the force of TRI necessary to balance the system is 3.46 kG (F_TRI_1_). Therefore, TRI torque is 0.1349 kGm (M_TRI_1_) and balances the gravitational torque (M_G_) of 0.1358 kGm (Fig. 1A). Balancing the system with an increased TRI arm of force from 0.039 m (r_TRI_1_) to 0.06 m (r_TRI_2_) (Fig. 1b), and with constant gravity (F_G_) and gravitational moment (M_G_), requires reducing the value of the TRI force to 2.275 kG (F_TRI_2_) (Fig. 1B). Despite the lower TRI force value, the torque due to the elongation of the arm by 0.021 m remains almost unchanged (the slight difference is due to measurement errors) and equal to 0.1349 kGm (M_TRI__2) (Fig. 1B,D), (Eq. 1).

1 $$\begin{array}{*{20}l} {M_{G} = \left( {1.205\;{\text{kG}} + 0.225\;{\text{kG}}} \right)*0.095\;{\text{m}} = 0.1358\;{\text{kGm}}} \hfill \\ {M_{TRI\_1} = 3.46\;{\text{kG}}*0.039\;{\text{m}} = 0.1349\;{\text{kGm}}} \hfill \\ {M_{TRI\_2} = 2.275\;{\text{kG}}*0.06\;{\text{m}} = 0.1365\;{\text{kGm}}} \hfill \\ {} \hfill \\ {M_{G} = M_{TRI\_1} = M_{TRI\_2} } \hfill \\ {{\mathbf{0}}{\mathbf{.1358}}\;{\text{kGm}} \approx {\mathbf{0}}{\mathbf{.1349}}\;{\text{kGm}} \approx {\mathbf{0}}{\mathbf{.1365}}\;{\text{kGm}}} \hfill \\ \end{array}$$

Based on the results of model tests and having the actual maximum values of muscle forces and the values of the arms of force, it is possible to calculate quite precisely which muscles have the highest rotational capacity. In the case of TRI, the maximum force is the sum of the forces of its three heads, i.e. SO: 945.95 kG. GM: 385.49 kG, and GL: 177.33 kG (Table 1) totaling 1508.77 kG. Knowing, that the TRI arm of the force is constant and equal to 0.04 m (Table 1), its total maximum moment of force for the upper ankle joint can be calculated, which equals 60.35 kGm (M_TRI_ = 1508.77 kG * 0.04 m) (Table 1).

In case of when TRI lost, for example, about 33% of its maximum strength due to weakness or paresis, its bending capacity, i.e. its torque, is equal to 40.43 kGm (1010.88 kG*0.04 m), and therefore decreased by approximately 19.91 kGm its physiological capabilities. Therefore, the foot takes on the characteristics of a talipes calcaneus. To compensate for the TRI flexion deficit rather than transposing other muscles, which is often performed in this situation (Supplement S.A), it can be increased the distance of the line of the Achilles tendon action from the axis of rotation of the upper ankle joint, which will increase the torque (flexing moment). This effect can be achieved, for example, by performing a Z-type osteotomy of the calcaneus in the sagittal plane. For a muscle weakened by 33%, it is enough to increase the arm of the force from 0.04 to 0.06 m by moving the calcaneal fragments apart, which will restore the TRI flexing ability to the physiological value (Eq. 2). To allow the fragments to move apart and prevent the development of a talipes cavus, a plantar fasciotomy should be performed.

2 $$\begin{array}{*{20}l} {{\text{F}}_{{{\text{TRI}}\_{1}}} = 1508.77\;{\text{kG}}\quad {\text{r}}_{{{1}\_{\text{TRI}}}} = \, 0.0{4}\;{\text{m}}} \hfill \\ {{\text{M}}_{{{\text{TRI}}\_{1}}} = {\text{F}}_{{{\text{TRI}}\_{1}}} *{\text{ r}}_{{{1}\_{\text{TRI}}}} = { 6}0.{35}\;{\text{kGm}}} \hfill \\ {{\text{F}}_{{{\text{TRI}}\_{2}}} = 1010.87\;{\text{ kG}} {\text{r}}_{{{2}\_{\text{TRI}}}} = \, 0.0{6}\;{\text{m}}} \hfill \\ {{\text{M}}_{{{\text{TRI}}\_{2}}} = {\text{F}}_{{{\text{TRI}}\_{2}}} *{\text{ r}}_{{{2}\_{\text{TRI}}}} = { 6}0.{65}\;{\text{kGm}}} \hfill \\ {} \hfill \\ {{\text{M}}_{{{\text{TRI}}\_{1}}} = {\text{ M}}_{{{\text{TRI}}\_{2}}} } \hfill \\ {{\mathbf{60}}.{\mathbf{35}}\;{\text{kGm }}\sim {\mathbf{60}}.{\mathbf{65}}\;{\text{kGm}}} \hfill \\ \end{array}$$

The operational increase in TRI torque, in addition to the dynamic effect, also strengthens the passive pressure of the foot to the ground. A similar effect is achieved by using orthopedic shoes in TRI paresis in which the heel is extended backward thus, the increased lever arm compensates for the weakened TRI flexing force during walking^19^.

The TRI arm of the force, TRI muscle force, and TRI muscle torque relationship are shown in Fig. 1C,D. The extent to which increasing the TRI arm of the forces can affect the torque that this muscle exerts on the upper ankle joint is shown on Fig. 1C. Figure 1D show that to maintain the rotational capacity of the TRI at a constant level, the TRI force must decrease as the length of the arm of the force increases.

#### Relationship between, the strength of the tibial posterior and the fibularis longus and their arms of the forces on the upper ankle joint level

**Figure 2:**
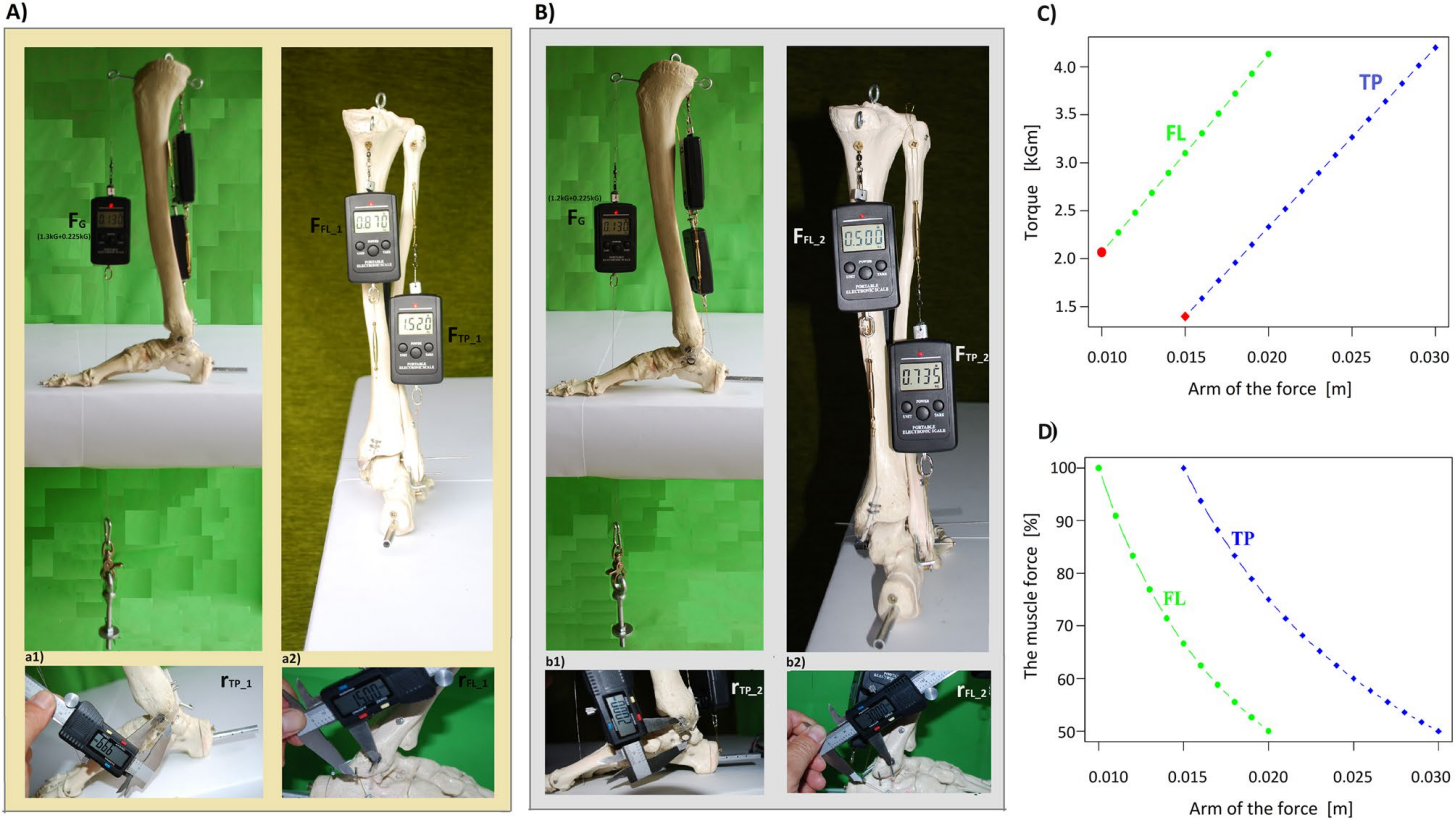
Balancing the moment of gravity with the sum of the torques of tibial posterior (TP) and fibularis longus (FL). The strength (force) of TP and FL at a constant gravity (F_G_ = 0.130 kG + 0.225 kG), the arm of gravity (r_G_ = 0.095 m), and the moment of gravity (M_G_ = F_G_*r_G_) on the upper ankle joint level. (**A**) In conditions similar to physiological ones, in which the arms of the forces for TP is 9.99 mm ~ 0.01 m (r_TP_1_), (F_TP_1_ = 0.87 kG), and for FL it is 0.015 m (r_FL_1_), (F_FL_1_ = 1.52 kG). (**B**) when the arm of the TP force is increased to a distance of 0.02 m (r_TP_2_), (F_TP_2_ = 0.5 kG), and FL at a distance of 0.03 m (r_FL_2_), (F_FL_2_ = 0.735 kG). (**C**) The relationship between the muscle torques and the length of the arms of the muscle forces at a constant value of the muscle forces for TP and FL. The red dot (for FL) and red square (for TP) stand for the physiological values of the torques for the physiological arms of forces. (**D**) The relationship between the forces of the muscles and the length of the arms of forces at a constant muscle torque for TP and FL.

In the experiment, the gravitational moment is balanced by the sum of the moments of two flexor muscles, i.e. tibialis posterior (TP) and fibularis longus (FL) (Fig. 2).

In conditions close to physiological, the arms of the forces of the TP and FL are 0.01 m (r_TP_1_) and 0.015 m (r_FL_1_), respectively, on the upper ankle joint level, and the forces of these muscles are 0.87 kG (FTP_1) and 1.52 kG (F_FL_1_) (Fig. 2A). When the arms of the forces are doubled to 0.02 m for TP (r_TP_2_) and 0.03 m for FL (r_FL_2_), for the sum of muscle force moments to balance the constant gravitational moment (MG), the value of muscle forces must be reduced. The force generated by TP in the model is 0.5 kG (F_TP_2_), and the force generated by FL is 0.735 kG (F_FL_2_), (Fig. 2B), (Eq. 3). Despite the lower value of the TP and FL forces, the sum of their torques because of the extension of the arms of the forces is unchanged. The small difference in the results obtained is due to the imperfections of the model.

3 $$\begin{array}{*{20}l} {M_{G} = \left( {0.130\;{\text{kG}} + 0.225\;{\text{kG}}} \right)*0.095\;{\text{m}} = 0.0337\;{\text{kGm}}} \hfill \\ {M_{TP\_1} = 0.870\;{\text{kG}}*0.01\;{\text{m}} = 0.0087\;{\text{kGm}}} \hfill \\ {M_{FL\_1} = 1.52\;{\text{kG}}*0.015\;{\text{m}} = 0.0228\;{\text{kGm}}} \hfill \\ {M_{TP\_1} + { }M_{FL\_1} = 0.0315\;{\text{kGm}}} \hfill \\ {M_{TP\_2} = 0.5\;{\text{kG}}*0.02\;{\text{m}} = 0.01\;{\text{kGm}}} \hfill \\ {M_{FL\_2} = 0.735\;{\text{kG}}*0.03\;{\text{m}} = 0.02205\;{\text{kGm}}} \hfill \\ {M_{TP\_2} + { }M_{FL\_2} = 0.03205\;{\text{kGm}}} \hfill \\ {} \hfill \\ {M_{G} = M_{TP\_1} + { }M_{FL\_1} = M_{TP\_2} + { }M_{FL\_2} } \hfill \\ {{\mathbf{0}}{\mathbf{.0337}}\;{\text{kGm}} \approx {\mathbf{0}}{\mathbf{.0315}}\;{\text{kGm}} \approx {\mathbf{0}}{\mathbf{.03205}}\;{\text{kGm}}} \hfill \\ \end{array}$$

Moving from the level of model research to the level of analysis of muscle moments of the weight-bearing human foot (Table 1), it can be calculated the actual maximum rotational capacity of the muscles and their effect on the foot. The maximum moments of force for the physiological course of the FL and TP thrust lines concerning the upper ankle joint are 1.50 kGm (M_FL_1_) and 2.07 kGm (M_TP_1_), respectively. Their flexing effect on the upper ankle joint is relatively small compared to TRI, but increasing their torques by increasing the arms of the forces can compensate for the slight weakening of the TRI. E.g. after doubling the values of the force arms, the torques for TP and FL are 4.133 (M_TP_2_) and 2.997 (M_FL_2_), respectively. In the case of such a change in the arms of the forces of the TP (r_TP_2_ = 0.02 m) and FL (r_FL_2_ = 0.03), could be compensated the weakening of the slight (5.9% of total force) TRI flexing force (F_TRI_1_ = 1419.75 kG. M_TRI_1_ = 56.79 kGm) (Eq. 4).

4 $$\begin{array}{*{20}l} {M_{TP\_1} = 206.66\;{\text{kG}}*0.01\;{\text{m}} = 2.07\;{\text{kGm}}} \hfill \\ {M_{FL\_1} = 99.92\;{\text{kG}}*0.015\;{\text{m}} = 1.50\;{\text{kGm}}} \hfill \\ {M_{TRI\_1} = 1508.77\;{\text{kG}}*0.04\;{\text{m}} = 60.35\;{\text{kGm}}} \hfill \\ {M_{TP\_1} + { }M_{FL\_1} + M_{TRI\_1} = 63.92{ }\;{\text{kGm}}} \hfill \\ {M_{TP\_2} = 206.66\;{\text{kG}}*0.02\;m = 4.133\;{\text{kGm}}} \hfill \\ {M_{FL\_2} = 99.92\;{\text{kG}}*0.03\;{\text{m}} = 2.997\;{\text{kGm}}} \hfill \\ {M_{TRI\_2} = 1419.75\;{\text{kG}}*0.04\;{\text{m}} = 56.79\;{\text{kGm}}} \hfill \\ {M_{TP\_2} + { }M_{FL\_2} + M_{TRI\_2} = 63.92\;{\text{kGm}}} \hfill \\ {} \hfill \\ {M_{TP\_1} + { }M_{FL\_1} { } + { }M_{TRI\_1} = M_{TP\_2} + { }M_{FL\_2} + { }M_{TRI\_2} } \hfill \\ {63.92\;{\text{kGm}} = 63.92\;{\text{kGm}}} \hfill \\ \end{array}$$

The distancing of the line of the muscle action from the axis of the upper ankle joint of the TP and FL muscles leads to an increase in the tonus of these muscles and, as a "side effect", significantly affects the lower ankle joint. The inversion effect caused by TP on the hindfoot and the eversion effect caused by FL on the forefoot is increased, thus deepening the longitudinal arch of the foot. TP increases the inversion of the heel, which shifts the line of the TRI action towards the medial side (de-valgus effect).

The arms of the forces, muscle forces, and muscle torques relationship for TP and FL is shown in Fig. 2B,C. The extent to which increasing the TP and FL arms of the forces can affect the torques that these muscles exert on the upper ankle joint is shown in Fig. 2C. Figure 2D shows that to maintain the rotational capacity of the muscles at a constant level, the force of the muscles must decrease as the length of the arms of the forces increases.

#### Relationship between, the strength of the tibial posterior, the fibularis longus, and the triceps surae and their arms of the force on the upper ankle joint level

**Figure 3:**
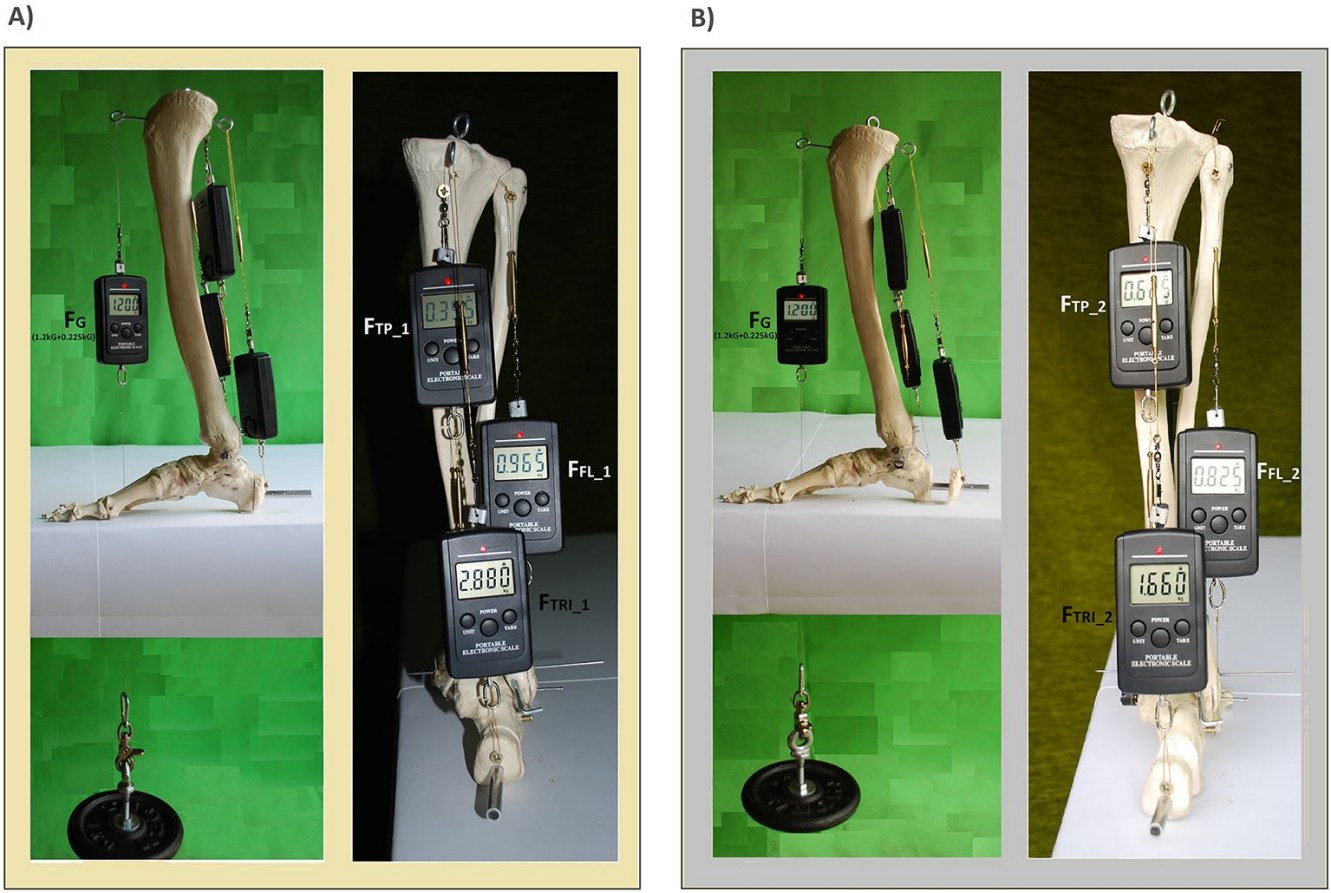
Balancing the moment of gravity with the sum of the torques of tibial posterior (TP), fibularis longus (FL), and Triceps surae (TRI). The strength (force) of TP, FL, and TRI at a constant force of gravity (F_G_ = 0.120kG + 0.225kG) and the gravity arm (r_G_ = 0.095 m), and the constant moment of gravity (M_G_ = F_G_*r_G_). (**A**) In conditions similar to physiological ones, in which the arms of the forces for TP and FL are 0.01 m (r_TP_1_) and 0.015 m (r_FL_1_) respectively, and for TRI is 0.0039 m (r_TRI_1_). (**B**) When the TP arm of the force is increased to a distance of 0.02 m (r_TP_2_), FL to a distance of 0.03 m (r_FL_2_), and TRI to a distance of 0.06 m (r_TRI_2_).

In the experiment to balance the constant moment of gravity were used the torques of three flexor muscles, i.e. tibial posterior (TP), fibularis longus (FL), and triceps surae (TRI) (Fig. 3).

In conditions similar to physiological conditions, the arms of the forces of TP, FL, and TRI are 0.01 m (r_TP_1_), 0.015 m (r_FL_1_), and 0.039 (r_TRI_1_), respectively, on the upper ankle joint level. The forces of these muscles are 0.395 kG (F_TP_1_), 0.965 kG (F_FL_1_), and 2.88 kG (F_TRI_1_), respectively (Fig. 3A). When the arms of the forces increase to the values of 0.02 m (r_TP_2_), 0.03 m (r_FL_2_), and 0.06 m (r_TRI_2_), the value of muscle forces decrease to balance the established, constant moment of gravity (M_G_). The force generated by TP in this model system is 0.625 kG (F_TP_2_), the force generated by FL is 0.825 kG (F_FL_2_) and TRI is 1.66 kG (F_TRI_2_) (Fig. 3B) (Eq. 5).

5 $$\begin{array}{*{20}l} {M_{G} = \left( {1.20\;{\text{kG}} + 0.225\;{\text{kG}}} \right)*0.095\;{\text{m}} = 0.13537\;{\text{kGm}}} \hfill \\ {M_{TP\_1} = 0.395\;{\text{kG}}*0.01\;{\text{m}} = 0.00395\;{\text{kGm}}} \hfill \\ {M_{FL\_1} = 0.965\;{\text{kG}}*0.015\;{\text{m}} = 0.014475\;{\text{kGm}}} \hfill \\ {M_{TRI\_1} = 2.88\;{\text{kG}}*0.039\;{\text{m}} = 0.11232\;{\text{kGm}}} \hfill \\ {M_{TP\_1} + { }M_{FL\_1} + M_{TRI\_1} = 0.1307\;{\text{kGm}}} \hfill \\ {M_{TP\_2} = 0.625\;{\text{kG}}*0.02\;{\text{m}} = 0.0125\;{\text{kGm}}} \hfill \\ {M_{FL\_2} = 0.825\;{\text{kG}}*0.03\;{\text{m}} = 0.02475\;{\text{kGm}}} \hfill \\ {M_{TRI\_2} = 1.66\;{\text{kG}}*0.06\;{\text{m}} = 0.0996\;{\text{kGm}}} \hfill \\ {M_{TP\_2} + { }M_{FL\_2} + M_{TRI\_2} = 0.13685\;{\text{kGm}}} \hfill \\ {} \hfill \\ \begin{gathered} M_{G} = M_{TP\_1} + { }M_{FL\_1} { } + { }M_{TRI\_1} = M_{TP\_2} + { }M_{FL\_2} + { }M_{TRI\_2} \hfill \\ {\mathbf{0}}{\mathbf{.13537}}\;{\text{kGm}} \approx {\mathbf{0}}{\mathbf{.1307}}\;{\text{kGm}} \approx {\mathbf{0}}{\mathbf{.13685}}\;{\text{kGm}} \hfill \\ \end{gathered} \hfill \\ \end{array}$$

Moving from modeling studies to a specific clinical case with approximately 37% TRI paresis, the effect of distancing the line of the action of the TP, FL, and TRI muscles to compensate for the paresis can be considered. If the heel lengthening (as discussed in section “Forces and torques produced by the extrinsic muscles of the foot”) cannot fully compensate for the TRI's bending capacity (M_TRI_2_ = 56.79 kGm), distancing of TP, and FL can be included. Distance of the TRI action line by increasing the force arm from 0.04 (r_TRI_1_) to 0.06 m (r_TRI_2_) together with increasing the distance of the FL action line from 0.015 (r_FL_1_) to 0.03 m (r_FL_2_) and TP action line from 0.01 (r_TP_1_) to 0.02 m (r_TP_1_) it is possible to obtain close to physiological flexing moment of the upper ankle joint of 63.92 kGm, (Eq. 6).

6 $$\begin{array}{*{20}l} {M_{TP\_1} = 206.66\;{\text{kG}}*0.01\;{\text{m}} = 2.07\;{\text{ kGm}}} \hfill \\ {M_{FL\_1} = 99.92\;{\text{kG}}*0.015\;{\text{m}} = 1.50\;{\text{kGm}}} \hfill \\ {M_{TRI\_1} = 1508.77\;{\text{kG}}*0.04\;{\text{m}} = 60.35\;{\text{kGm}}} \hfill \\ {M_{TP\_1} + { }M_{FL\_1} + M_{TRI\_1} = 63.92{ }\;{\text{kGm}}} \hfill \\ {M_{TP\_2} = 206.66\;{\text{kG}}*0.02\;{\text{m}} = 4.133\;{\text{kGm}}} \hfill \\ {M_{FL\_2} = 99.92\;{\text{kG}}*0.03\;{\text{m}} = 2.997\;{\text{kGm}}} \hfill \\ {M_{TRI\_2} = 946.5\;{\text{kG}}*0.06\;{\text{m}} = 56.79\;{\text{kGm}}} \hfill \\ {M_{TP\_2} + { }M_{FL\_2} + M_{TRI\_2} = 63.92\;{\text{kGm}}} \hfill \\ {} \hfill \\ {M_{TP\_1} + { }M_{FL\_1} { } + { }M_{TRI\_1} = M_{TP\_2} + { }M_{FL\_2} + { }M_{TRI\_2} } \hfill \\ {{\mathbf{63}}{\mathbf{.92}}\;{\text{kGm}} = {\mathbf{63}}{\mathbf{.92}}\;{\text{kGm}}} \hfill \\ \end{array}$$

### Relationship between the tibial anterior strength and its arm of the force on the upper ankle joint level

The main muscle that balances the backward leaning of the center of gravity from the axis of the upper ankle joint is the tibial anterior (TA). This muscle develops a great of torque, greater than the sum of the torques of the remaining foot extensors (Table 1), making it an effective antagonist of the triceps surae. Weakness or paralysis of TA causes numerous deformities of the foot, whose treatment involves serious surgical intervention in the anatomical structures of the foot (Supplement S.B).

Balancing the system when the center of gravity is leaned backward from the axis of the upper ankle joint results in the distancing of the TA inserts. The lamina pedis moves away from the lower leg (flexion position), and TA is in eccentric contraction. The flexor muscles are not involved in balancing the backward leaning of the lower leg. They are only in compensatory concentric contraction to ensure smooth movement.

**Figure 4:**
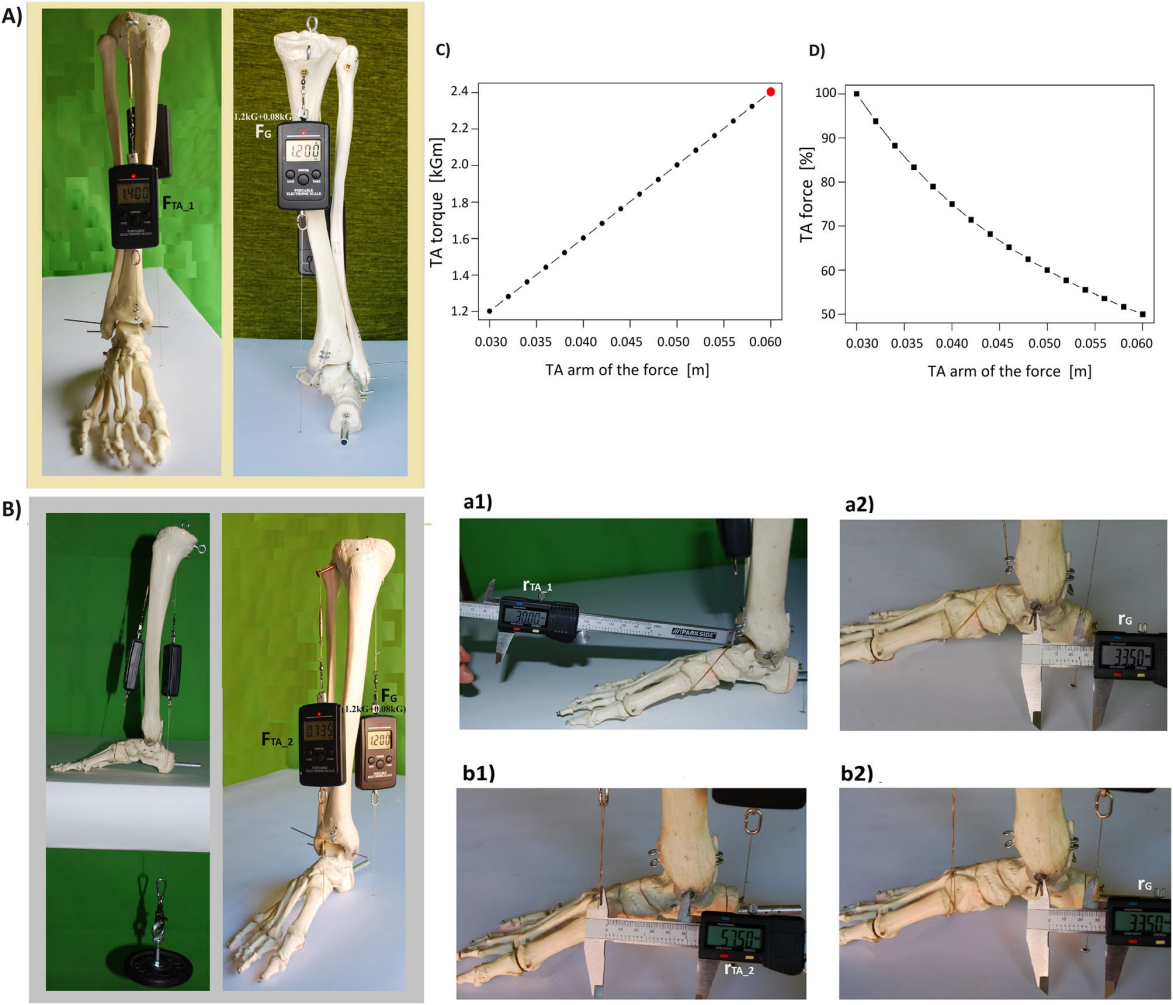
Balancing the moment of gravity by the tibial anterior (TA) torque. The strength (force) of TA at a constant value of the force of gravity (F_G_ = 1.2 kG + 0.08 kG), and moment of gravity (M_G_ = F_G_*r_G_ = 0.0428), on the upper ankle joint level. (**A**) in conditions similar to physiological ones, in which the arm of the force for TA is 0.03 m (r_TA_1_), (F_TA_1_ = 1.4 kG). (**B**) when the TA arm of the force is increased to 0.0575 m (r_TA_2_) by releasing a cable imitating the TA tendon from two hooks acting as extensor retinaculum (F_TA_2_ = 0.735 kG). (**C**) The relationship between the torque and the length of the arm of the force at a constant value of the force of the muscle weakened by 50%. As the arm grows, the torque increases to a value close to the physiological value (red dot). (**D**) The relationship between the force of the muscle and the length of the arm of force at a constant torque of a muscle weakened by 50%. The more weakened muscle the length of the arm of the force proportionally longer to maintain the physiological torque. (**a1**) Measurement of the TA arm of the force in a condition similar to the physiological one (r_TA_1_ = 0.03 m). b1) Measurement of the TA arm when the line of action of the muscle is moved away (r_TA_2_ = 0.0575 m). b1) Measurement of the arm of gravity without (**a2**) and with (**b2**) moving away from the TA line of action (r_G_ = 0.0335 m).

To achieve the balance of the system when the lower leg is leaned backward, the gravitational moment (M_G_ = F_G_*r_G_) must be balanced with the muscular moment (M_TA_ = F_TA_*r_TA_) (Fig. 4).

In conditions close to physiological, when the TA arm of the force is 0.03 m (r_TA_1_) (Fig. 4a1), the arm of gravity is 0.033 m (r_G_) (Fig. 4a2), and the fore of the gravity of leaning and loaded lower leg with the dynamometer is 1.28 kG (F_G_ = 1.2 kG + 0.08 kG), the TA force necessary to balance the system is 1.4 kG (F_TA_1_) (Fig. 4A). Therefore, the TA moment of force is equal to 0.042 kGm (M_TA_1_) and balances the moment of gravity (M_G_) with a value of 0.043 kGm. Balancing the system with an increased arm of the force of TA from 0.03 m (r_TA_1_) (Fig. 4b2) to 0.0575 m (r_TA_2_) (Fig. 4b1), and with constant force and gravitational moment (F_G_, M_G_), requires reducing the TA force to 0.735 kG (F_TA_2_). Despite the lower value of the TA force, the torque due to increasing the length of the arm of the force remains approximately the same and equals 0.0422 kGm (M_TA_2_) (Fig. 4B) (Eq. 7).

7 $$\begin{array}{*{20}l} {M_{G} = \left( {1.2\;{\text{kG}} + 0.08\;{\text{kG}}} \right)*0.0335\;{\text{m}} = 0.04288\;{\text{kGm}}} \hfill \\ {M_{TA\_1} = 1.4\;{\text{kG}}*0.03\;{\text{m}} = 0.042\;{\text{kGm}}} \hfill \\ {M_{TA\_2} = 0.735\;{\text{kG}}*0.0575\;{\text{m}} = 0.0422\;{\text{kGm}}} \hfill \\ {} \hfill \\ {M_{G} = M_{TA\_1} = M_{TA\_2} } \hfill \\ {{\mathbf{0}}{\mathbf{.04288}}\;{\text{kGm}} { } \approx { }{\mathbf{0}}{\mathbf{.042}}\;{\text{kGm }} \approx {\mathbf{ 0}}{\mathbf{.0422}}\;{\text{kGm}}} \hfill \\ \end{array}$$

Based on the results of model tests, having the value of the maximum force of TA and the value of the arm of the force, the torque can be calculated. In the case of TA, the maximum force is 80.16 kG (F_TA_1_), the arm of the force of TA is constant and equals 0.03 m (Table 1), so the maximum moment of force (torque) is 2.4 kGm (M_TA_1_ = 80.16 kG*0.03 m) (Table 1).

If TA, due to a 50% weakening of its maximum force, is unable to balance the moment of gravity (M_G_), it is enough to distance the TA action of the line from 0.03 to 0.06 m (r_TA_2_) from the axis of the upper ankle joint. It can be done by cutting extensors retinaculum at the level of the TA sector that causes the TA rotational possibilities will return to the norm (M_TA_2_ = 40.08 kG*0.06 m), (Eqs. 8). The release of the tendon must be combined with its shortening to adjust the proper tonus.

8 $$\begin{array}{*{20}l} {M_{TA\_1} = 80.16\;{\text{kG}}*0.03\;{\text{m}} = 2.4\;{\text{kGm}}} \hfill \\ {M_{TA\_2} = 40.08\;{\text{kG}}*0.06\;{\text{m}} = 2.4\;{\text{kGm}}} \hfill \\ {} \hfill \\ {M_{TA\_1} = M_{TA\_2} } \hfill \\ \end{array}$$

The TA arm of the force, TA muscle force, and TA muscle torque relationship is shown in Fig. 4C and D. The extent to which increasing the TA arm of the forces can affect the torque that this muscle exerts on the upper ankle joint is shown on Fig. 2C. Figure 2D shows that to maintain the rotational capacity of the TA at a constant level, the TA force must decrease as the length of the arm of the force increases.

## Discussion and conclusion

So far, the professional literature has not presented the real values of muscle forces and real values of the moments of forces acting on the foot only relative values of the muscle strength^1^. Simultaneously, many surgical techniques are proposed that are directly related to them^4^. Few studies provide only relative comparisons of torques at the foot level^6,8,10^. Most medical textbooks use the concept of muscle force to describe the action of muscles on the foot joints. Meanwhile, the effect of the real action of the muscle on the joints of the foot is the effect of rotational force, i.e. the torque (the moment of force) that the muscle can develop on a specific joint^3,6,9^. To date, considerations regarding neuromuscular surgery for the treatment of muscle paresis of the feet have focused on tendon transfer techniques based on an analysis of the approximate relative strength of the muscles^1^. The success of procedures depends largely on the intuition and many years of experience of surgeons who during the procedure react to the observed effects of their actions and adjust them to the desired effect^4^. Their effort is not based on hard biomechanical data of the foot. However, the professional literature shows progress in research of the musculoskeletal system. The concept of "muscle balance" had been in force for years, described in 1899 by Codivilla^20^ in tendon transfer surgery in connection with surgeries for muscle paralysis and paresis was rejected^1^. This was the result of analyses that suggested that the flexor muscles of the upper ankle joint are six times stronger than the extensor muscles of this joint. Therefore, it has been proposed in foot surgery to apply the principle of task appropriateness to be performed by a specific muscle transfer to maintain the physiological asymmetry of muscle strength.

Unfortunately, the results that transplant surgeons have relied on for years turn out to be, at least, imprecise in the light of our analyses. Silver's first error^1^ was that he concluded about the strength of the flexors and extensors of the upper ankle joint by including muscles that did not affect the upper ankle joint. He determined the percentage of muscle forces by including all external and internal muscles of the foot and then referred to the muscles acting only on the upper ankle joint. This disturbed the perception of the actual involvement of the flexors and extensors of the upper ankle joint and resulted in false estimates of the strength proportions of these muscles. Moreover, he did not include all flexor muscles in his estimates (specifically the tibial posterior and the fibularis longus and brevis). Meanwhile, based on our calculated maximum values of forces of all muscles (Table 1) acting on the upper ankle joint, it was determined that the strength of the flexor muscles alone is 12 times greater than that of the extensor muscles. Silver's important conclusion was that the proportions of forces of the intrinsic and extrinsic muscles of the foot in different individuals are approximately the same^1^, which was also noticed in studies of the muscles of the upper limb^21^. Silver's big discovery was the finding that the same forces of agonists and antagonists cannot be used during muscle transfers. because, as he proved, there is a significant asymmetry between them. However, it was a mistake not to take into account the key element in the action of muscles on joints, which is the distance of the lines of action of the muscles from the axis of rotation of the joints they act on i.e. "the arms of the forces". Taking into account the arms of the forces (based on our experiments), it has been shown that in the human foot, there is a 14.6-fold advantage in the rotational force of the flexors over the extensors of the upper ankle joint. This large predominance of the flexors results from the need to balance the moment of gravity during standing and walking and from the need to ensure gait propulsion. Our other observation, which contradicts previous reports is real rotation force of the tibialis anterior muscle. Commonly, the strongest extensor of the foot is considered the tibial anterior (TA), whose strength exceeds the sum of the forces of the other extensors of the foot^9,11^. Operating on the values of maximum muscle forces (Table 1), it was calculated that the sum of EHL, EDL, and FT muscle forces is 83.48 kG. This value exceeds the TA force value (F_TA_ = 80.16 kG). Therefore, what anatomists observe^9^, i.e. the advantage of the TA "force" over the sum of the “forces” of the remaining foot extensors, results from the rotational capacity of the TA (Table 1), whose torque is 2.40 kGm and exceeds the sum of the torques of the remaining foot extensors equal 2.16 kGm. The case above demonstrates how important is to compare "rotational force" (muscle torque), and not the absolute value of muscle force. The torque is an extremely important parameter, especially knowing that all movements in the foot are rotational movements^5^ and most tendons are away from the axis of rotation of the joint. To perform a rotational movement in this situation, a smaller value of muscle contraction force is needed than if the tendon is close to the axis of rotation of the joint. Hence, a muscle with less strength can successfully balance a muscle with greater strength because its arm of the force is correspondingly larger^22^. A muscle can develop the greatest force, but if the arm of the force is zero then there is no rotational action on that joint, only the articular congruence component is present. Surgical procedures based on changing the size of the arm of the muscle force can significantly reduce the scope of surgical interventions compared to classic muscle transfer methods. In the described approach, there is no need to create wide tendon tunnels and the risk of damage to the neurovascular bundle and the development of postoperative scarring may be reduced. In most cases of muscle transfers, they lose their original functionalso often undergo atrophy^3^. In the proposition presented in the paper, moving away the line of action of the tendon from the axis of rotation of the joint does not cause the risk of muscle atrophy. The approach proposed in this paper is not free from limitations. For example, you cannot extend the force arm of the triceps calf muscle twice because it would result in foot deformation. Similarly, there are limitations to the separation of the lines of action of the tibial posterior and peroneus longus muscles. This is related to the movement of the forks, which, if extended too much, would resist on the plantar side of the foot. Determining the limit of the length of the force arms of individual muscles will be possible after performing computer simulations and a series of experiments with the implants used. Considering the above, the current method of surgical treatment and kinesiotherapy of the foot should be revised, including primarily the moment of force in the description of the action of muscles on the joints. Detailed knowledge of foot biomechanics with specific values of muscle torques and tissue properties can be used to develop simulation models, e.g. based on 3D CT, precisely calculating the range of correction for personalized treatment. This will allow surgeons to be better prepared for the procedure and reduce the likelihood of side effects.

## Methods

**Figure 5:**
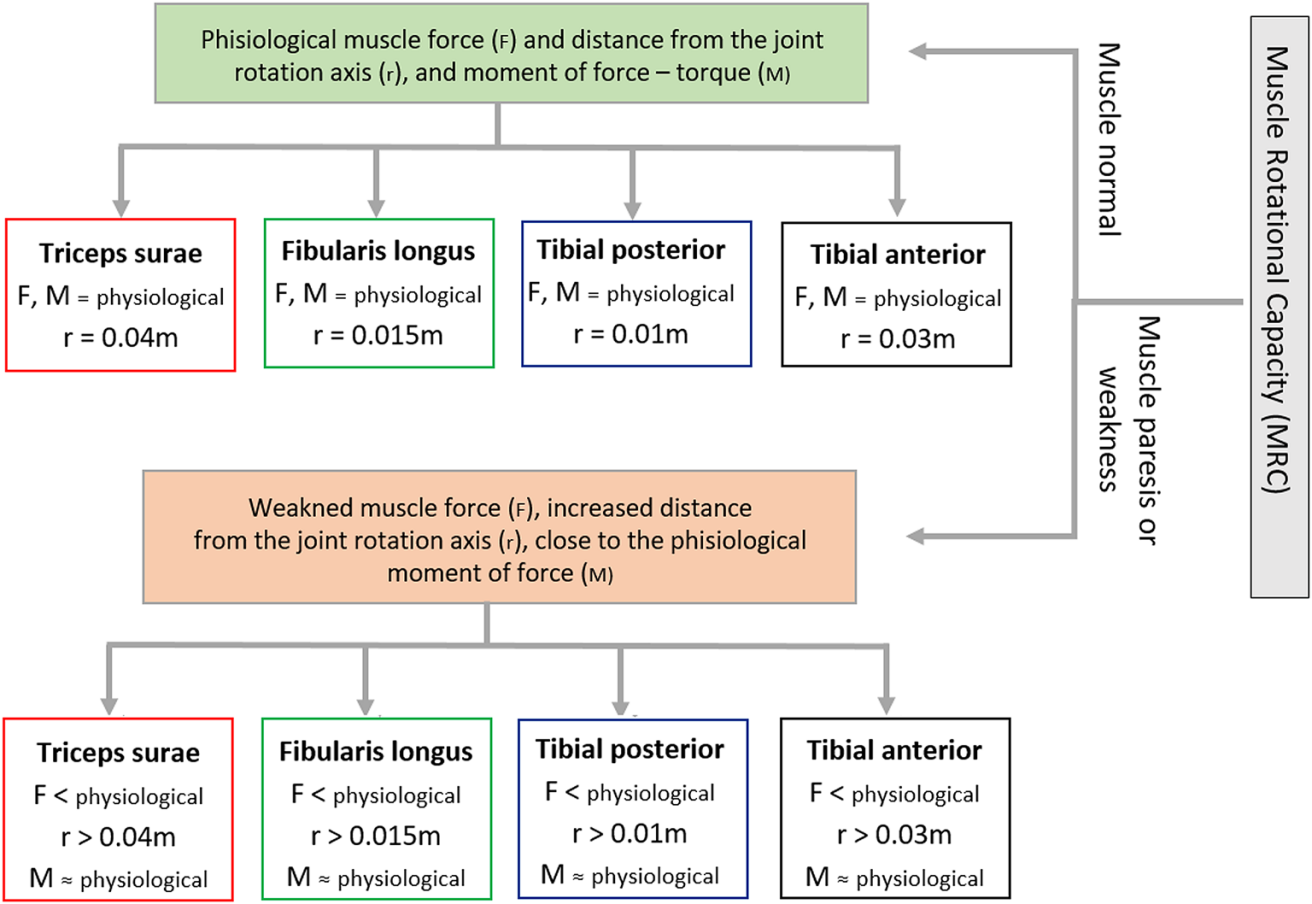
A diagram showing the relationship between the strength and the force arm of the external muscles of the foot and their torque in a normal situation and when the muscle force is weakened. By increasing the arm of the force, the rotational capacity of weakened muscles can be increased and even bring it closer to the physiological value of torque.

The research results regarding the muscle arms of the forces^15^ and data regarding the length of muscle fibres, muscle mass, and muscle pinnation^2^ were used to calculate the torques of extrinsic muscles acting the upper ankle joint. Based on experiments, the "physiological" situation is shown, when the muscles operate with physiological force and their distance from the joint rotation axis is within the norm, and which parameters should be manipulated when the muscle is weakened and wants to approach the physiological state (Fig. 5).

### Forces generated by the extrinsic foot muscles

Based on the extended definition of PCSA^23^ (the original version did not take into account muscle pinnation^24^), including data on muscle mass, muscle pinnation (pinnation angle—the angle of inclination of the fibres to the tendon), muscle tissue density and length muscle fibre^2^, physiological cross-sections were calculated for each extrinsic foot muscle according to formula (9)^2^.

9 $$PCSA=\frac{mucle \, mass \, [g]*cos\Theta }{\rho \left[\frac{g}{cm3}\right]*fibre \, length \, [cm]}$$

where PCSA—physiological cross-sectional area, Θ is pinnation angle, ρ is muscle density (1.06 g/cm^3^).

Since that muscle force is proportional to PCSA, and knowing that the muscle force is 10 kG/1 cm^2^ PCSA^9,25^ (other authors report a value of 30 N/cm^2^^26^), the maximum contraction force (Fm) of extrinsic muscles was calculated according to formula (10).

10 $${F}_{m}=10 kG* PCSA\left[{{\text{cm}}}^{2}\right]$$

where F_m_—muscle contraction force.

The force of muscle contraction was expressed in two units: kilogram-force [kG] (1 kG = 9.81*1N) and newton [N] (1N = 1 kG/9.81). It was assumed that all sarcomeres are effectively activated simultaneously.

### The muscle torques generated at the upper ankle joint level

The muscle torque calculations were based on formula (11) for torque definition.

11 $${M}_{m}={F}_{m}*r*{\text{sin}}(\alpha )$$

where M_m_—the moment of muscle force (muscle torque), F_m_—the force of the muscle, r—the arm of the force, and (α)—the angle between the vector of F_m_ and r.

Excluding a few it was assumed that analysed tendons of the foot muscles run at right angles to the axis of joint rotation^16^. The shortest distance from the line of action of the muscle to the axis of rotation of the joint is called the arm of the force (r). In this system, the force arm (r) is positioned perpendicular to both the force vector (F_m_) and the joint rotation axis. In such conditions, the muscle develops the greatest torque (M_m_) (sin(90°) = 1). It was assumed that the line of action of the muscle is limited to the section between the end insertion and the point of the structure that changes the direction of action of this muscle. The value of the force of the muscle and the length of the arm of the force were used to calculate of moment of force for each muscle whose line of action crosses the upper ankle joint. The calculated moments of force were expressed in kilogram-force-meters [kGm] and Newton-meters [Nm] (1 Nm = 9.81*1 kGm).

### The research model of external muscles acting on the upper ankle joint

#### Model construction

A physical model was created to simulate the leaning of the human body supported by the foot and demonstrate the forces of the muscles acting on the upper ankle joint taking into account the force of gravity. The experiment aimed to demonstrate the balance of the entire system, in which the moments of muscles balance the moment of gravity. The model was used to measure the forces of the muscles and moments of forces in normal conditions and when the course line of muscle action was in distance above the norm. The research model consists of the lower leg connected with the lamina pedis by a hinge. It imitates the action of the upper ankle joint when the lower leg is leaning (forward or backward), and the lamina pedis is based on the ground.

In the model:

- All joint connections within the lamina pedis have been stiffened to expose the hinge connection between the lamina pedis and the lower leg.
- The axis of the upper ankle joint was made of a steel rod and set physiologically, i.e. at an angle of 10º plantar deviation in the frontal plane and 6º backward deviation in the horizontal plane. There are six low-resistance micro-bearings mounted on the axis, which ensure rotation of the talus block relative to the distal part of the lower leg.
- Steel cables and turnbuckles were used to simulate the course of muscle tracts, which were connected to dynamometers demonstrating the strength of muscle tracts. Teflon tubes, metal grommets, and rods, as well as a carbon fibre ring, reproduced the muscle strings along with the change in the direction of the muscle action lines. The ends of metal cables imitate muscle attachments (attached by micro-screws). The dynamometers and the cables connected to them did not touch other dynamometers or the lower leg bones.
- The projection of the centre of gravity was simulated by attaching to the lower leg a loaded metal cable with a dynamometer.
- The distance of the lines of muscle strings was simulated depending on the anatomical conditions of individual muscles concerning the axis of the upper ankle joint:

- Triceps calf—a metal tube was inserted into the proximal part of the transversely cut calcaneus, on which the calcaneal tuberosity with the attachment of the cable imitating the Achilles tendon was moved as if on a guide, which made it possible to distance the line action of the muscle from the axis of the upper ankle joint.
- Tibial anterior—increased distance the line action of the muscle was achieved by releasing the cable imitating a tendon from two hooks being in the role of an extensor retinaculum.
- Tibial posterior—increased distance was obtained by adding a carbon fibre ring with a diameter of 0.01m to the natural rotation arm, which is the medial malleolus.
- Fibularis longus—increased distance was achieved by the implementation of a metal rod bent in an L-shape to the top of the lateral malleolus, which is a natural arm of the torque.

The distancing of the muscle strings from the axis of the upper ankle joint was supplemented by adjusting the length of the cables, maintaining their tension to guarantee an unchanged arm of the gravitational force value when the lower leg was leaned forward and backward.

The starting point for examining the body's leaning relative to the upper ankle joint was the Basic Balance Point (BBP), which is obtained when the projection of the centre of gravity falls on the axe of the upper and lower ankle joint^16^. Then the muscular moments and the gravitational moment are zero because the arms of the force arms are equal to zero.

#### Determination of the weight of the body leaning beyond the basic balance point

To calculate the moments of muscle forces and the moment of gravity concerning the axis of the upper ankle joint, the weight of the lower leg leaning beyond the Basic Balance Point (BBP) forward and backward in the sagittal plane was determined (Fig. S1). BBP is the point where the axes of the upper and lower ankle joints intersect, on which the projection of the centre of gravity falls, causing both extensors and flexors of the upper ankle joint as well as invertors and evertors of the lower ankle joint are excluded from the contractile activity. The weight of the lower leg leaning forward is 0.155 kG, and the lower leg with the dynamometer is 0.255 kG, while the weight of the lower leg leaned back along with the dynamometer is 0.08 kG. The determination of the weight of the lower leg tilted backwards was omitted due to the negligible difference (within the margin of error) in the weight of the lower leg itself comparing the lower leg with a dynamometer. The distance of the projection of the centre of gravity from the axis of the upper ankle joint in the case of an example leaning of the lower leg forward was 0.095 m from the axis of the upper ankle joint and in the case of a backward leaning of the lower leg 0.0335 m. In further measurements, the value of the arms of gravity was kept constant when the lower leg was leaning forward and backwards in the sagittal plane. Measurements of the arms of the forces were made using a caliper and in cases with difficult access to the axis of the upper ankle joint, a specially constructed upright frame and dynamometers (Ruhhy® 0.005–40 kg) were used.

### Data processing

The numerical data was processed using R^27^ and the Graphics library. It was used to develop torque versus force arm length graphs to demonstrate how an increase in the force arm length affects the torque value.

### Supplementary Information

Supplementary Information.

## Data Availability

All data generated or analysed during this study are included in this published article [and its supplementary information files].
