# Supplementary material for: Torques in the human upper ankle joint level and their importance in conservative and surgical treatment
Source: Sci Rep. 2024 Mar 29;14:7525. doi: 10.1038/s41598-024-57698-4 (PMC10980753; doi:10.1038/s41598-024-57698-4)
Supplement: Supplementary file 1 — Supplementary Information. [file 41598_2024_57698_MOESM1_ESM.docx]

Supplement


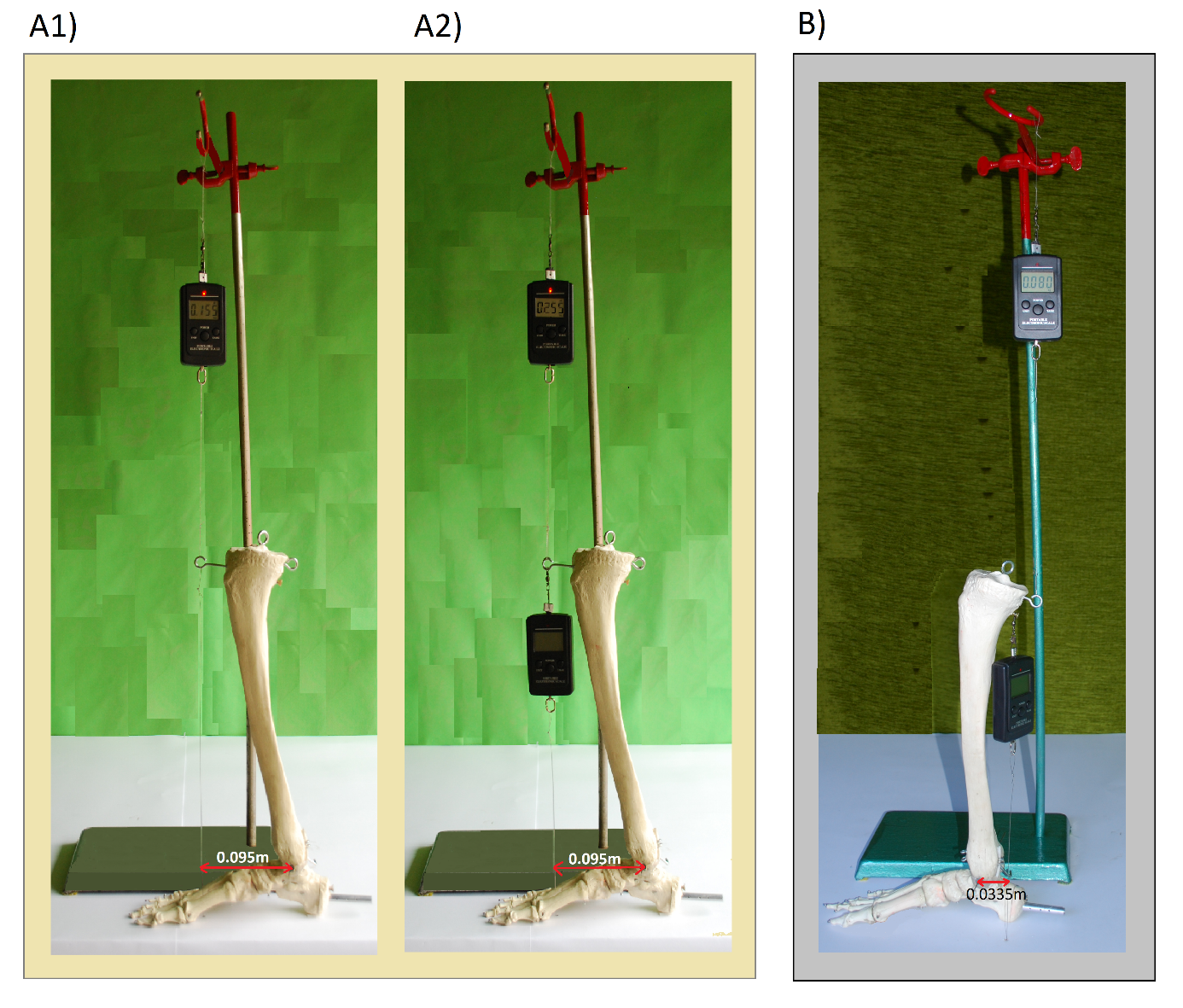
**Figure S.1.** Weighing the lower leg leaning beyond the basic balance point. A1) leaning of the lower leg forward. A2) leaning of the lower leg together with a dynamometer forward. B) leaning of the lower leg together with
a dynamometer backward.

**S.A)**

The widespread operation of orthopedic surgeons in the scope of operating muscle paresis is limited to restoring the flexing force of three-headed triceps surae through transfers of other flexors not being in paresis, e.g. tibial posterior (TP) and fibularis longus (FL) [1] [2]. The main idea is to strengthen a weakened or inactive muscle with another muscle. These types of treatments were developed based on many years of experience of surgeons who confirmed the effectiveness of this type of procedure through experiments. They were based mainly on their intuition and regarding the healthy side without biomechanical knowledge (e.g. the distance of the line of the muscle action from the axis of the joint rotation, absolute muscle forces) for a specific surgical procedure due to the lack of data.

Below is an example of the treatment of triceps surae paresis by strengthening its function with the transplantation of the tibial posterior (TP), and fibularis lungus (FL) muscles to the calcaneus [1].

In triceps surae paresis, a deformity known as talipes calcaneus gradually develops. In this pathology, the gait becomes limping due to only the heel contacting the ground and the lack of propulsive action of the flexors. In order to facilitate the support phase, flexor grafts are used in the area of the calcaneal bone to support the function of the triceps surae which is in paresis. Moreover, they are protected against overload by the front locking of the upper ankle joint. In the initial phase of the disease, there are no changes in the heel position yet. In the initial phase of the disease, when the heel position has not changed yet, in order to prevent the least disturbance of dynamic balance, TP is transplanted on the medial side, and FL on the lateral side. The ends of the tendons are dragged through the heel canal, crossing them and sewing them together so that both form one loop. The peripheral stump of FL is sewn into the fibularis brevis (FB) tendon, and the peripheral stump of a TP is sewn into the tibial anterior (TA) tendon. In the case of paralysis of the muscles for transfer mentioned above, the flexors of flexor digitorum longus (FDL), and flexor hallucis longus (FHL) are used. It has been observed that the transplantation of both fibularis muscles while the active tibial posterior (TP) leads to talipes varus, and while the active FHL leads to the dorsal setting of the metatarsal bones [1] [2].

**S.B)**

Weakness or paralysis of TA causes weakness or loss of dorsiflexion and inversion, resulting in a slowly progressive talipes equinus and talipes cavus or varying degrees of planovalgus deformity. Extensor digitorum longus (EDL), which usually supports dorsiflexion in an attempt to support TA becomes excessively active, which results in hyperextention of the proximal phalanges and lowering of the heads of the metatarsal bones (claw toes). This type of deformity is treated surgically by lengthening the Achilles tendon with capsulotomy and transferring fibularis longus (FL) to the base of the second metatarsal bone. To prevent postoperative dorsal bunion deformity, the fibularis brevis (FB) tendon is sutured to the peripheral stump of the fibularis longus (FL) (FB is sutured to the stump of FL to prevent a dorsal bunion). Alternatively, the inserts of EDL can be moved back to the dorsal side of the midfoot, to ensure active dorsiflexion [2]. In each case, this involves serious interference with the anatomical structures of the foot, often related to this, that the muscle was transferred, compensating for the tasks of the paralyzed/paretic muscle loses its physiological course and previous function.

**References**

[1] A. Gruca, Chirurgia ortopedyczna - tom III, II, Państwowy Zakład Wydawnictw Lekarskich, Warszawa, 1993.

[2] F.M. Azar, J.H. Beaty, S.T. (S. T. Canale, W.C. (Willis C. Preceded by: Campbell, Campbell’s operative orthopaedics, 14th ed., Elsevier, 2020.
